# “We’re all in it together”: uniting a diverse range of professionals and people with lived experience within the development of a complex, theory-based paediatric speech and language therapy intervention

**DOI:** 10.1186/s40900-025-00738-8

**Published:** 2025-06-19

**Authors:** Lucy Rodgers, Nicola Botting, Natalie Abdo, Meriem Amer-El-Khedoud, Emma Baker, Sophie Franks, Dave Harford, Patrycja Salimi-Tabar, Laura Temple, Ros Herman

**Affiliations:** 1 Department of Language and Communication Sciences, City St George’s, London, UK; 2 Sussex Community NHS Foundation Trust, Brighton, UK; 3 Barts Health NHS Trust, London, UK; 4 Sheffield Children’s NHS Foundation Trust, Sheffield, UK; 5 West Sussex County Council, Worthing, UK; 6 Faculty of Health, Education and Life Sciences, Birmingham City university, Birmingham, UK; 7 Ethnic Minority Achievement Service, Brighton and Hove City Council, Brighton, UK; 8 Brighton and Hove Inclusion Support Service, Brighton and Hove City Council, Brighton, UK

**Keywords:** PPI, Co-design, Intervention development, Speech and Language therapy

## Abstract

**Background:**

It is increasingly commonplace to involve relevant professionals and people with lived experience within healthcare research. Although valuable case studies regarding such involvement exist, there is currently a paucity of case studies highlighting the professional and personal impacts of uniting a diverse group of professionals and people with lived experience within the development of new, theory-based interventions. The aim of this paper is to provide insight into the impacts of involving a diverse range of individuals, unified within a single steering group, within the development of a new, theory-based, paediatric speech and language therapy intervention (“Supporting Words and Sounds” - SWanS). By describing the involvement process in detail and providing our personal insights, we hope our recommendations will be of use to future healthcare researchers.

**Main body:**

Our project steering group consists of two people with lived experience (an adult with Developmental Language Disorder-DLD, a parent of a child with DLD), three specialist NHS speech and language therapists (including one university lecturer with equality and diversity expertise), and two individuals working in the education sector (a specialist teacher and a bilingual educational support worker). Group members have been involved across the 4 phase intervention development process. Tools such as the PiiAF (Public involvement impact Assessment Framework) have guided our personal and professional reflections on our individual experiences of being in a diverse steering group responsible for developing a new and complex theory-based intervention.

**Conclusion:**

We found that having a diverse range of people unified in a singular intervention development steering group had unexpected benefits. Learning from each other has enriched professional practice and developed individuals’ confidence in terms of playing an active role in research. Our structured reflection has implications for future intervention development research, by highlighting that the provision of a safe, supportive space and nurturing of shared values is key when involving a diverse range of parties. Such contexts promote sustained involvement and therefore have longer term implications for increasing the relevance of the research for those it is aiming to help.

**Supplementary Information:**

The online version contains supplementary material available at 10.1186/s40900-025-00738-8.

## Abstract (plain language summary)

Some children find it difficult to learn to talk for no obvious reason. They may have difficulties learning words (language) *or* saying sounds (speech). Some children have both speech *and* language difficulties. We need to develop a speech and language therapy intervention to help them. This is complex. This is because the intervention needs to be based on theory. This includes linguistic theories about how sounds and words develop in young children.

Our steering group has helped develop a new theory-based intervention called SWanS (“Supporting Words and Sounds”). Together, we have helped make the intervention the best it can be. Our group consists of people with lived experience (an adult with language difficulties, a parent of a child with speech/language difficulties). Our group also consists of two educational professionals and three speech and language therapists. In this paper, we are reflecting on our personal and professional experiences of being involved in the group. We want to share the potential benefits of bringing together people with different backgrounds when developing a theory-based intervention. We used tools such as the PiiAF (Public involvement impactAssessment Framework) to help us reflect.

We found it important to have people from different backgrounds in our steering group. We all feel more confident in taking part in research after being in the group together. We have also learnt a lot from each other’s experiences. We have taken these learnings into our personal and professional lives. This was possible due to the group being a safe and supportive space.

**Supplementary Information:**

The online version contains supplementary material available at 10.1186/s40900-025-00738-8.

## Background

### Introduction

It is widely recognised that meaningful engagement with key stakeholders, including professionals and people with lived experience, is fundamental to developing new healthcare interventions [1]. Intervention steering groups are responsible for providing oversight, direction, and personal insights within the intervention development process [2]. Although published examples of relating to the *process* of intervention co-design are increasing, there is still a need for more nuanced examples regarding the personal experiences of steering group members to serve as learning opportunities [3]. This commentary reports on our personal reflections of bringing together a diverse range of professionals (NA, EB, LT, M A-K, P S-T) and people with lived experience (SF, DH) within the development of a new theory-based complex intervention for pre-school children with co-occurring features of speech sound disorder (SSD) and developmental language disorder (DLD). We have named the intervention “SWanS” (Supporting Words and Sounds).

There are two key complexities to the co-design approach. Firstly, the academic researchers (LR, NB, RH) have had to balance *authentic* co-design with building intervention theory; the two may not necessarily align [4]. Specifically, there is the challenge of introducing theory into co-design whilst simultaneously prioritising the voices of people with lived experience [5]. Secondly, because of the incremental nature of theory building (adopted in the current study), the intervention development process has required intensive input from a diverse steering group of both people with lived experience and relevant professionals from the very onset.

We hope that the reflections from our steering group (NA, EB, SF, DH, M A-K, LT, P S-T) and resulting recommendations will be of use to intervention developers from across healthcare, who may be considering similar co-design structures in the future.

This paper starts with LR (lead academic researcher) providing some background to the project, i.e. why a new intervention is being developed, methods to facilitate steering group involvement, and impacts of involving the steering group on the intervention. We (the project steering group), then reflect on the impacts of involvement for us personally and provide principles and corresponding recommendations for future research.

### Why develop a new intervention?

It is estimated that approximately 7.6% of 4-year-olds present with features of Developmental Language Disorder (DLD) [6]. DLD is characterised by difficulties in learning and using new words, forming sentences, and/or understanding spoken language [7]. Features of DLD in the pre-school years can cause frustration, as the child is often unable to communicate their needs effectively [8]. Speech Sound Disorder (SSD) is a separate childhood-onset condition which affects approximately 3.4% of 4-year-olds [9]. The most common sub-group of SSD, phonological SSD, is characterised by difficulties in learning and producing speech sounds [10]. This often results in the child not being understood by those around them, also leading to frustration [11]. Although DLD and SSD can occur in isolation, they often overlap, with approximately 40.8% of children with SSD also having features of DLD [9]. A co-occurring SSD/DLD profile in the pre-school years is associated with long term negative outcomes in literacy and language [12] and has downstream consequences for mental health [13, 14].

There is considerable evidence to indicate that pre-school speech and language therapy (SLT) interventions can alleviate, or even resolve, pre-school features of SSD *or* DLD [15, 16]. Within UK NHS services, these interventions often involve the child having sessions with a speech and language therapist (SaLT) in clinic, with the SaLT supporting the child’s parent to continue with activities at home [17]. To date, there are few evidence-based interventions available to SaLTs working with children with a co-occurring profile, and which are implementable within routine clinical practice [18]. Therefore, we are developing a new intervention for pre-school children with a co-occurring SSD/DLD profile, called SWanS (Supporting Words and Sounds),

### The intervention development process

There is no one size fits all approach to developing interventions, with each prospective intervention having its own unique qualities [1]. The MRC guidance for developing and evaluating complex interventions was used to plan the intervention development phases, with the lead academic researcher (LR) choosing to focus on solving key uncertainties- i.e. what questions do we need to answer, before moving on to the next stage in the development process? This resulted in 4 broad phases:

Phase 1- Systematic review: **what is the best available evidence?**

Phase 2- Survey: **what does current clinical practice look like?**

Phase 3-e-Delphi: **what are the agreed core elements for a new intervention?**

Phase 4- Co-design: **how can we enhance the acceptability of these core elements?**

This intervention development process is theory-based with linguistic theory, behaviour change theory, and implementation science being integrated into one or more of the study phases. Originally, co-design elements were predominantly incorporated into phase 4, where the focus of the project shifts from the identification of core intervention elements (based on theory) to operationalisation. However, even within predominantly theory-based interventions, excluding voices of people with professional and lived experience from the outset could limit the impact and potential applicability of the intervention further down the line [5]. Therefore, in response to pre-project patient and public involvement (PPI) feedback, the lead academic researcher (LR) decided that a combined theory-partnership approach to intervention development was required [19]. This involved setting up a project steering group to oversee and input into each study phase. Unlike the co-design work at phase 4, being in the steering group would be a relatively large time investment, spanning all 4 phases of the project. One benefit of having two options for involvement was that individuals could choose to be involved at a level they felt most comfortable with [20]. For example, parents who would be unable to commit to the steering group could still take part in co-design activities in phase 4. A thorough description and evaluation of the phase 4 co-design work is currently being written up in a separate article.

The decision to form a combined steering group consisting of a variety of different professionals and people with lived experience was based on pre-study PPI and clinician engagement work. In this work, both parents of children with SSD/DLD and SaLTs conveyed a strong preference for joint working.

### Summary (aims)

At the start of the intervention development process, the lead academic researcher’s (LR) over-arching aims for forming a steering group were to:

1. Integrate a diverse range of voices into the development of a new theory-based complex speech and language therapy intervention.
2. Resolve ‘key uncertainties’ relating to the 4 intervention development project phases as a team.

However, the objective of this commentary is for our steering group (NA, EB, SF, DH, M A-K, LT, P S-T) to shine a light on our *personal* experience of being involved in a diverse steering group within the development of a theory-based intervention.

Therefore, the aims for this paper are to:

1. Provide individual reflections on our experiences, framed around the topics of making a difference, learning from others, and expanding own learning.
2. Provide individual reflections on barriers and accessibility.
3. Share our group’s recommendations for future research, which are based on our reflections.

Although often overlooked, personal experiences of PPI and professional engagement are important for understanding the potential benefits and harms of different approaches [3]. Therefore, although the reflections of researchers can be included in commentary papers, we have made the decision to fully centre the reflections of steering group members.

We hope that we can provide some useful learning points which can be applied to future research aiming to bring together a diverse group of individuals within theory-based intervention development.

## Main text

### Setting up and structure

#### Group responsibilities

Due to the specific needs of this 4-phase intervention development project, some aspects of the steering group responsibilities were set by the lead academic researcher (LR) prior to recruitment. Including, that the group would meet regularly every 3–4 months (with longer, frequent meetings towards the end of the project) so that their input could be integrated at each research phase. In response to pre-project PPI feedback, the lead academic researcher also decided that steering group members would need to be able to access online meetings, to enable people from across the UK to regularly attend. Key steering group responsibilities for each project phase can be viewed in Table 1.

**Table 1:** Project phases and key tasks for the steering group to support with

Phase and aim	Methods	Main responsibilities/tasks to support with
**PHASE 1** Identify the best available evidence for joint SSD/DLD interventions	Systematic review	Create the extraction form. Discuss and prioritise key findings.
**PHASE 2** Identify how SaLTs are currently supporting pre-schoolers with a co-occurring SSD/DLD profile	Quantitative online survey	Develop survey questions. Discuss and prioritise key findings.
**PHASE 3** Consensus with specialist SaLTs on the core intervention elements	e-Delphi with specialist SaLTs	Develop statements for round 1 of the e-Delphi based on findings from phase 1 (systematic review) and 2 (survey). Revise statements not achieving consensus in round 1 of the e-Delphi.
**PHASE 4a** Enhancing acceptability of core intervention elements	Community PPI with parents from under-represented communities.	Refine methods to optimise the accessibility for parents taking part. Develop verbal/written/visual intervention description and supporting materials. Decide on refinements to the intervention based on parent feedback. Co-design PPI evaluation questions to ask parents taking part.
**PHASE 4b** Enhancing the feasibility of the intervention protocol	Interviews with speech and language therapists	Develop the interview schedule. Discuss findings, turn into a ‘plan of action’ for the next stage in the development-evaluation process.

#### Recruiting to the steering group

Public involvement within healthcare research may widen disadvantage and further marginalise under-represented groups if additional steps are not taken to include them [21]. As children from low income backgrounds face additional barriers to accessing SaLT services whilst being disproportionately affected [22], and non-English speaking children and families from global majority backgrounds are typically under-represented in practice-based SaLT research [23], it was deemed essential by the lead academic researcher to include professionals with experience of working within minoritised communities. Therefore, professional steering group members were required to work within communities with high areas of deprivation and/or communities with a high level of non-English speakers and/or global ethnic majority groups, as indicated by the ONS (Office of National Statistics) ranking for their geographical area [24, 25]. The lead academic researcher aimed to recruit a minimum of one parent of a child with DLD/SSD, and an adult with lived experience of accessing paediatric SaLT services. Due to project needs, the academic researchers widened the inclusion criteria for people with lived experience, who could be from minoritised or non-minoritised backgrounds. Professionals sought included SaLTs working in different geographical locations, and a minimum of one professional working within education. Representation of professionals from the education sector was deemed important due to the close working relationships between NHS SaLTs and teaching staff [17].

A purposeful recruitment approach was taken. Firstly, an information sheet (additional file 1) was shared via e-mail and a Facebook post through the DLD charity NAPLIC. The information sheet was also distributed amongst the lead researcher’s pre-existing networks, including parents who had taken part in the pre-project PPI work. Interested individuals then had a discussion with the lead researcher over telephone or video, giving them the opportunity to ask questions, get further clarification, and decide if they wished to proceed.

By the end of the recruitment process, the lead academic researcher (LR) had formed a steering group consisting of:

Public partners:

- A parent of a child with DLD, and speech and language therapy advocate (DH).
- An adult with DLD who accessed NHS SaLT as a child, with experience of supporting children in education settings (SF).

Professional partners:

- Three specialist NHS SaLTs working in disadvantaged areas of the UK (EB, NA, M A-K), including one with specialist EDI (Equality, Diversity and Inclusion) expertise (M A-K).
- A specialist early years teacher supporting children with DLD/SSD in pre-school education settings (LT).
- An educational bilingual support worker for the Brighton and Hove Ethnic Minority Achievement Service (EMAS), with professional experience of supporting bi/multi-lingual children with SSD/DLD, and lived experience of raising young children in a trilingual environment (P S-T).

#### Initial stage: setting up

Once individual steering group members had been recruited, a period of 3–4 months was dedicated to ‘planning for involvement’ [26] prior to commencing research-specific activities. This time was used for the lead academic researcher to have in depth individual discussions with each steering group member about their level of involvement, access needs and communication preferences, roles/responsibilities, and their personal aims for taking part in the steering group. The researcher used a discussion guide (additional file 2) based on the 6 principles of the UK standards for public involvement [27]. The guideline of “inclusive opportunities” was a particular focus, for example by establishing preferred means of communication between whole group meetings (e-mail, text, face calls), all of which varied between the different group members (one member preferred face to face video calls, two members preferred text messaging, four members preferred e-mail). Aspects of ‘working together’ were facilitated through the co-creation of documents such as a ‘shared principles’ ethos (additional file 3) and ‘role outline’ (additional file 4). These were developed with steering group members to set and manage expectations for the group work ahead. In the first whole group meeting the lead researcher recapped and summarised what was discussed in the individual meetings, with an emphasis on getting to know each other and finding common ground. For example, each group member took it in turns to share why they felt SaLT was important. Importantly, this was a shared passion of all steering group members, regardless of background.

#### Subsequent meetings and structure for involvement

Whole group and individual meetings with the lead academic researcher continued every 3–4 months. An online scheduling tool [28] was used to identify the most convenient time and date for group members to meet. If a professional member (NA, EB, M A-K, LT, P S-T) was unable to attend a session, their availability was prioritised for the subsequent session, to ensure that they did not miss two group meetings in a row. The availability of our two group members with lived experience (DH and SF) was also prioritised by the lead academic researcher, to ensure that there were two people with lived experience at all meetings. When a member was unable to attend a session, the lead academic researcher met up with them separately to go over what had been covered. The individual meetings between each steering group member and lead academic researcher largely consisted of ‘checking in’ (i.e. seeing how the steering group experience was going for that person), discussing any queries/issues of particular interest to the individual, and introducing discussion points which would be covered in the following group meeting. The over-arching timeline for the steering group meetings and activities can be found in Table 2.

**Table 2:** Timeline for steering group activities

Timeframe	Activity (monitored using the impact log)	Focus
10/2022-11/2022	Recruitment	
10/2022-1/2023	Individual meetings	Preparing for involvement. Introduce shared principles and role outline. Identify barriers/enablers to participation and personal aims for individual group members.
1/2023	Whole group + individual meetings 1	Group cohesion-shared values and appreciation of SLT. Recap purpose of steering group and the project, including the shared principles. Introduce phase 1 (systematic review).
3/2023	Whole group + individual meetings 2	Extraction form for phase 1. Brainstorm benefits/disadvantages of different intervention deliverers. Recap communication between meetings.
6/2023	Whole group + individual meetings 3	Phase 1 findings reflection. Finalising wording for phase 2 (survey). Brainstorming how to optimise accessibility of phase 4a for parents from diverse backgrounds.
10/2023	Whole group + individual meetings 4	Refining the phase 4a involvement strategy. Review of steering group impact so far. Confirm content for conference 1 slides. Review/agree abstract for conference 2.
12/2023	Whole group + individual meetings 5	Comparing/contrasting phase 1 and phase 2 findings. Mapping potential actions on discussion boards. Introduction to phase 3 (e-Delphi).
3/2024	Whole group + individual meetings 6	Conference 2 poster content. Review of impact so far. Guest talk from PhD fellow *guest speaker* on child/family language use during daily routines, with Q&A after.
6/2024	Whole group + individual meetings 7	Phase 3 (e-Delphi) statement generation. Behaviour change technique selection. Feedback on how conference 2 presentation was received.
9/2024	Whole group + individual meetings 8	e-Delphi update and revision of statements which did not achieve consensus. Generation of questions to ask parents when evaluating the phase 4a PPI strategy.
11/2024	Whole group + individual meetings 9	Finalising phase 4a PPI strategy. Reviewing/refining the intervention description to go with the video for phase 4a. Brainstorm of our over-arching reflections on being part of the steering group.
2/2025	Whole group + individual meetings 10	Update on phase 4a. Recap previous reflections and Public Involvement Impact Assessment Framework (PiiAF) record card items. Reflect on personal goals and confirm article content.
*Final meetings (planned)*		
*May 2025*	*Whole group + individual meetings 11*	*Refinements to core intervention elements following phase 4a (parent PPI)*,* finalising content for phase 4b (SaLT interviews).*
*July 2025*	*Whole group + individual meetings 12*	*Implications of phase 4b findings*,* discussion re: next steps*,* what to focus in the first intervention trial.*
*September or October 2025*	*Whole group + individual meetings 13*	*Celebration*,* staying in contact*,* what’s next?*

#### Tools for reflection

The researchers and steering group used tools for reflection throughout the intervention development process to aid ongoing reflexivity and the recording of impacts on the intervention being developed, as well as the intervention development process. For example, the lead academic researcher used an impact log [29] to draw a direct link between steering group activities and the impact of any resulting decisions made. The impact log included a description of the discussion topic, actions/timeframe resulting from the discussion, and then a ‘longer term impact’ section to return to after the actions had taken place. The impact log was added to after every whole group and individual meeting. For example, following individual discussions with the lead academic researcher, details of changes to the data extraction form in phase 1 (systematic review) were recorded in the log. Group members with lived experience added in items relating to family (DH) and child (SF) perspectives, and professional group members added in items relating to NHS clinical practice (EB, MA-K, NA) and education (LT, PS-T). We (the lead academic researcher and steering group) then returned to the log after the systematic review was completed. We recorded that the ‘longer term impact’ was the potential generalisation of systematic review findings for people with relevant lived/professional experience [30, 31]. Secondly, the lead academic researcher used the impact log to record any potential barriers or supports to being part of the steering group raised by individual group members. For example, one group member reported that it was difficult to remember what was discussed previously; in response to this, the lead academic researcher included a recap section at the start of each whole group meeting. Having such actions written down with a timeframe for completion helped to make sure they were actioned in a timely manner.

Joint dissemination activities as the project progressed, such as the writing up of journal articles, also provided valuable opportunities for reflection and evaluation of impact. Although the GRIPP 2 short form was used as a reporting tool [30, 31], the content of the GRIPP form for each project phase was also a point of discussion within steering group meetings. These discussions provided the space for steering group members to reflect on a tangible example of the positive impacts they had made so far, before moving onto the next project phase. Similarly, conference presentations, co-authored by group members, also provided opportunities for reflection. Three examples include: (1) our reflections on involving a diverse range of stakeholders in the systematic review process [32], (2) our co-designed strategy for involving people from marginalised communities within phase 4 of the project [33], and (3) SF and LR’s reflections and recommendations on involving adults with neurodevelopmental conditions within paediatric research [34]. Although many impacts on the intervention being developed were a result of group working, different group members had individual contributions because of their differing expertise and backgrounds. Examples are given below:

1. SF discussed the importance of therapy being “fun”, as this is what made it a good experience for her as a child. Therefore, we have made the intervention activities flexible and based on the child’s interests, rather than having a rigid SLT agenda.
2. When identifying potential ‘behaviour change techniques’ (BCTs) for the SLT to use with the parent as part of the intervention, DH explained how one of the BCTs listed could be harmful and promote distrust between the parent and the SLT. This BCT was therefore not included in the e-Delphi (phase 3).

In addition to the above, the Public involvement impact Assessment Framework [35] (PiiAF) was used to guide the reflection process and to generate the recommendations presented within the next section of this article. The PiiAF is a structured approach to reviewing the impact of public involvement activities, based on four key themes: values, approaches to involvement, how the involvement fits into the wider research, and practical considerations.

The PiiAF record card (additional file 5), based on these four areas, was developed by the lead academic researcher by using discussion points recorded within the impact log following the initial discussions with each steering group member.

Important reflection points highlighted include:

1. Barriers/accessibility to taking part in the steering group activities;
2. Personal aims and learning journeys (making a difference, learning from others, expanding own learning).

### Reflections and recommendations

In preparation for writing this article, within an online, whole group meeting we (NA, EB, SF, DH, M A-K, LT, P S-T) split into two subgroups and brainstormed positives and areas for improvement from our steering group experience on an interactive whiteboard [36] (additional file 6). An interactive whiteboard was selected as the best means of recording the discussion due to it being visual (e.g. colour coded grids), with each steering group member being able to individually add to the board using their personal login, both during and between whole group meetings. These discussions were led by steering group members, without the presence of the lead academic researcher. We highlighted positives such as the opportunity be part of research, learning from other group members’ lived experiences and perspectives, and gaining confidence over time. Areas for improvement included facilitating regular contact with other group members, initial uncertainty about what would be involved/the value of individual contributions and sometimes finding it difficult to find the time in a busy week for meetings. After the whole group meeting, the lead academic researcher then asked each steering group member to reflect on their individual experiences, based on the areas raised in the PiiAF (making a difference, learning from others lived experience, expanding own learning, barriers/accessibility). Depending on the needs and preferences of each group member, some wrote their accounts alone (EB, MA-K) whilst others wrote them during an individual meeting with the lead academic researcher (DH, LT, NA, PS-T, SF).

## Making a difference

### Dave (public partner)

I absolutely feel like I’ve made a difference and have contributed to the research on several levels. Getting to meet the team and understand their experiences has been humbling- and to think they’ve taken away some of my contributions is fantastic. I think I’ve given the other group members a unique perspective on what it’s like on the other side of the fence. We have all learnt from each other. My contributions have not just impacted the research, but also the professionals’ practice and the families they work with. That’s amazing.

### Emma (professional partner- NHS)

As a speech and language therapist working with children in this age range with DLD and SSD, it has been very rewarding to be involved in research that will have a meaningful and practical application for this group of children and their families. I have had the opportunity to share some of the stories and experiences of children and families I have worked with and have tried to keep their needs in focus when discussing the different stages of developing the intervention. As a lot of research in the speech and language therapy world is difficult to apply in NHS clinical practice, it has been refreshing to be involved in research where co-design, and practical considerations such as acceptability for parents, children and clinicians, are central to its design.

### Laura (professional partner- education)

It’s difficult to find something that supports the needs of children with overlapping DLD/SSD features. To have an intervention designed with them in mind, it’s great. For professionals, it makes us more aware of their needs, as you have that added element of frustration and awareness when the child also has SSD. Thinking of how to incorporate their needs into an intervention is key, and we are giving them an opportunity they won’t necessarily have had. It’s not just about speech and language; this intervention will help in every area of learning and relationships too. I’ve enjoyed advocating for the children. Having worked with children and their parents, I have a sense of how difficult life can be for them. Raising awareness has been important for me too.

### Meriem (professional partner- NHS)

I truly believe that, as a group, we have made a significant impact in considering how to support children with DLD/SSD. I was able to draw on my own clinical experience and conversations with student speech and language therapists to explore areas that are not always addressed in other interventions. Having the other members of the steering group to discuss ideas with has played a huge role in the difference we have made as a team.

### Natalie (professional partner- NHS)

It’s been fulfilling to draw on my clinical experience of DLD/SSD interventions and put that knowledge into some of the intervention content. My experience and input has been framed by the context of the experiences of the other group members. It’s not just about our experience and expertise as individuals, but how as a collective we have come together to make the intervention as good as it can be. Having Dave and Sophie in the group has also been helpful when thinking about how the intervention will unfold.

### Patrycja (professional partner- education)

From my point of view, I am representing the voice of families who have English as an Additional Language (EAL) and people who do not always access information or think that their voice matters in the therapy process. These are families who may refuse or delay support because they don’t feel confident. I did feel ‘should I be here?’ at the start of our project because of the strong experience of the other group members. I was also worried about not accurately representing people from my background properly. As time went on my role and how I was making a difference became clearer. I was glad to contribute some good points to the discussion as EAL families are now a big part of the everyday work of a SaLT.

### Sophie (public partner)

I tend to put myself down because of my understanding. Having DLD is hard and I do worry about not being able to understand or express myself. I’ve obviously made a difference to the project by talking about my childhood experiences of therapy. It helps when Lucy tells me how what I’ve said has made a difference. I also feel like I’m making a difference when I’m able to contribute to the discussions.

## Learning from others lived experience

### Dave (public partner)

As a parent of a child with DLD, it’s been interesting to see the perspective of an adult with DLD. I already knew what SaLTs do day-to-day, but I now understand more about the challenges they face in terms of awareness of speech and language therapy and impact on self-esteem. I’ve also learnt that research is a lot of work!

### Emma (professional partner- NHS)

Being in the steering group has been a fantastic opportunity to meet and collaborate with professionals and individuals with lived experience of DLD and SSD from across the country. Through the group meetings and breakout rooms, we have been able to have some in-depth discussions, covering topics that wouldn’t normally be discussed in my clinical work. This has given me some valuable insights, particularly from Sophie and Dave’s own experiences, but also helping me to consider some of the barriers that families might encounter in my own practice. It has been fantastic to meet Sophie and Dave in person in national events/ conferences that we have attended. It has also been really valuable to consider differences in service delivery from professionals from different parts of the UK.

### Laura (professional partner- education)

It’s been great working with Sophie and Dave. I’ve had lots of chats with Dave, it’s interesting to talk to him. I’d like to keep in contact with him and perhaps invite him to one of our parent groups. He’s a really great link - he’s been there, his son has been there. I’ve found Sophie’s perspective fascinating and practically helpful in terms of strategies that she said help her. I now use these in my own practice. Because I’m not used to working from this angle, collaborative work with people with lived experience on a project has been enriching. It’s also been interesting to work with professionals from across the country. We all have varied perspectives and ways of working.

### Meriem (professional partner- NHS)

Working with Dave and Sophie has been invaluable - learning from those with lived experience has been such a positive and insightful experience for me. I hope to carry that learning into my day-to-day practice and teaching. Equally, Patrycja’s, Natalie’s, Emma’s, and Laura’s experiences have been just as important. It really makes me think about the bigger picture and how we each play a role in the puzzle of supporting children with DLD/SSD. Our different perspectives come together to ensure we can support them in the best possible way.

### Natalie (professional partner- NHS)

I think having a parent advocate in the group and someone with DLD has been extremely important. It’s been interesting in smaller group discussions to reflect on certain topics and what their experiences have been. I have clinical experience of supporting parents and people with lived experience, but not within the context of working collaboratively on a big project. It’s been valuable learning what’s been helpful and difficult for them. They have also shown me that it’s not all negative! Using their positive experiences to inform the intervention, as well as my own practice, has been great.

### Patrycja (professional partner- education)

Meeting Sophie has been incredible - she is the first adult I have met with DLD. My conversations with her are something I will take into my future work with families. I can reassure parents of children with DLD that their child will be able to have a fulfilling life when they grow up, with the right strategies in place.

### Sophie (public partner)

It’s been interesting talking to Dave and hearing about what life is like for those who have family members with DLD.

## Expanding own learning

### Dave (public partner)

I never thought I’d be in something like research because of my academic levels - but I’ve learnt that life experience is just (if not more) valuable than a piece of paper. This project has given me the confidence to take part in research and I think that is largely because the group has been so supportive. I would definitely do it again. When I first said yes to taking part in this project I would have laughed at the idea of how far I’ve come.

### Emma (professional partner- NHS)

It has been fascinating to learn about the depth and scope of developing a new intervention and gaining a detailed step-by-step insight into the 4 phases of this research study. I have learnt a lot about the value of involving a wide variety of perspectives in shaping the intervention in a way that will be as functional and accessible as possible for children and families. I found it particularly interesting to learn more about the processes of developing surveys and the e-Delphi study. I have recently been developing a new pathway in our service and I have been able to apply some of this learning in creating a survey for parents and speech and language therapists.

### Laura (professional partner- education)

What’s been unique about this experience is seeing how a complex intervention development project is put together. It’s been presented in a way where we can understand and not feel lost in. I now understand how you would go about putting together such a big piece of intervention development work. It’s been important that these processes have been made clear and explicit to us, with recaps in each meeting.

### Meriem (professional partner- NHS)

I have really enjoyed seeing this group come together and learning how to best manage a diverse research team. Lucy has been clear in outlining the aims of the research and has guided us through each phase, making it easier to follow - especially with our busy schedules.

### Natalie (professional partner- NHS)

Learning about the research process has been interesting, from aspects of the intervention context through to linguistic theory and behaviour change. Having *guest speaker* come in to talk about her research on use of language in daily routines was interesting and informative for my clinical work as well as well as the intervention. I particularly enjoyed being involved in the e-Delphi process and seeing it from an ‘insider’ perspective, in contrast to being a research consumer.

### Patrycja (professional partner- education)

This project has been a fantastic experience, and a first for me. It’s been great speaking to so many other different professionals as well as Dave and Sophie. I’m not a SaLT but at the same time I’ve been able to take what I’ve learnt and discuss it with the families I work with and my colleagues. I can see how much preparation goes into therapy and feel I am now better able to reassure families who are worried about the therapy process. I tell them that their voice matters.

### Sophie (public partner)

It’s been interesting working with other SaLTs and Lucy. It’s nice to extend my circle of people that know about DLD. I find research interesting as it’s not clear why people have DLD. Anything we can do to help is important.

## Barriers/accessibility

### Dave (public partner)

The polls have been helpful because there is more than one option of time/date to meet, the flexibility of that is brilliant. The main issue for me was shift work, but there was nothing much that could be done about that. Recaps at the start of group meetings helped a lot. It would have been helpful to have had a WhatsApp group from the very start, so we didn’t have to wait 3 months to contact each other.

### Emma (professional partner- NHS)

As a busy clinician and mum of 3 teenagers, it can be tricky to find time for additional activities, however I felt the frequency of meetings was generally very manageable and there was a lot of flexibility for meeting times, particularly for the 1–1 meetings. Everyone in the group is so friendly and approachable that I have felt comfortable to ask questions and to work with other members in small breakout rooms. All information has been presented clearly with re-caps at the start of every meeting and plenty of opportunities for questions and clarification when needed.

### Laura (professional partner-education)

The polling scheduling software has been really good. It’s helped that there has been a lot of flexibility with times. Due to this I’ve been able to work my steering group commitments around my new baby and family life. I found it helpful to still be cc’d into emails when on my maternity leave, so I was kept on the loop. Lucy also did a helpful ‘catch up’ session with me when I returned.

### Meriem (professional partner-NHS)

Having a range of available dates to meet was helpful. My main challenges were time commitments, and unfortunately, I wasn’t able to attend all the meetings. However, recaps from Lucy and one-on-one meetings have been helpful, as they allowed me to stay in the loop and still contribute to the research.

### Natalie (professional partner- NHS)

Even though it’s an additional time commitment, I really think that having the 1:1s has been important for keeping things fresh in the mind. I’ve found the wide option of dates and times helpful. Recent unforeseen workload and team changes within my NHS trust have made my steering group responsibilities more difficult to balance recently. As a clinician you cannot always predict how busy you might be 2 years into the future, however I did find the outline of the time commitment given at the start helpful.

### Patrycja (professional partner- education)

My main barrier was being unsure of my suitability for the group at the start. I tend to feel some impostor syndrome in the things I do. I felt my confidence grow with time- everyone in our group listens and is respectful. The group is so friendly and even though we’re scattered all over the place, when we get together it works. Everyone has persevered with the project, no-one has dropped out, and that is largely down to the group being so friendly. It’s also really helped that there are options of times to meet and that the availability of others has been explicit through the doodle poll.

### Sophie (public partner)

Keeping up with the conversations has been hard sometimes. Reminders to put on captions helps. It was also helpful working in smaller groups, although I can feel embarrassed if I can’t contribute. It’s helped that the other group members are kind and get me to join in. I explain that I find it a bit tricky sometimes and they understand.

### Summary of reflections

In a whole group meeting we split into two sub-groups (without the lead academic researcher present) to develop summaries of our individual reflections. Sub-group one focused on ‘making a difference’ and ‘learning from others’ lived experience’ and sub-group two focused on ‘expanding own learning’ and ‘barriers and accessibility’. First, each sub-group member read the reflections individually and made their own notes. Sub-group members then came together for group discussion and developed summaries. Following the meeting, the four summaries were then shared via e-mail by the lead academic researcher to all members of the steering group, for final member checking. The summaries of our reflections are provided in table 3, below.

**Table 3:** Summary of steering group reflections

Focus	Summary
Making a difference	Each steering group member felt that they made an impact on shaping the intervention. This impact was more meaningful and effective because of the collaborative approach taken, whilst acknowledging individual expertise and lived experience. Group members cared deeply about ensuring that the intervention was practical, accessible, and applicable in real life settings.
Learning from others’ lived experience	Everyone participating in the steering group was enthusiastic and passionate about the subject. The lived experience of public partners helped professional members of the group to better understand the real life impact of DLD/SSD, and it helped shape the intervention being developed. Collaboration between professionals from across the country also helped enrich discussions around the intervention.
Expanding own learning	Many members of the steering group described an increase in confidence in engaging in research. All group members acknowledged and valued the contributions of those with lived experience and practitioners commented on ways in which this has informed their practice. Steering group members felt that they had learnt a lot about the detailed process of completing research and developing an intervention. For some, this has directly impacted their clinical work. Members valued the opportunity to learn from one another’s experiences and broaden their understanding of DLD. The clear aims and structured intervention development process helped with this learning.
Barriers/accessibility	Group members appreciated flexibility in time slots and having potential meeting dates/times on a poll, as this helped them to plan around busy schedules. Having recaps, e-mail updates, and 1:1 meetings with the lead academic researcher helped to keep members up to date if they were unable to attend a meeting. Group members found the frequency of meetings manageable, with it helping to have a clear schedule from the start. No-one left the group, demonstrating a good level of accessibility. Positive group dynamics helped members to feel safe to contribute and ask questions.

## Key principles and practical recommendations

The reflections from each steering group member highlight that bringing together a diverse group of individuals within the development of a complex, theory-based intervention can have positive personal and professional consequences. These positive consequences can be both within and beyond the remit of the actual project itself. However, to enable this personal growth and fulfilment to occur, the right conditions and working environment are essential. In a whole group meeting we identified and agreed on 5 key principles arising from our reflections. We brainstormed potential recommendations based on these key principles (additional file 7). The lead academic researcher (LR) then e-mailed a summary of recommendations for member checking. The key principles and final recommendations are within Table 4 below.

**Table 4:** Key principles and practical recommendations

Key point	Practical recommendations
Group cohesion, kindness and respect is foundational.	Establish shared values with individuals, and then as a group, at the very start of the process. If disclosed (and with consent), ensure that all group members are aware of how to support neurodivergent group members (e.g. members with developmental language disorder, dyslexia, autism, ADHD). Allocate time for icebreakers and informal discussion at the start of each group session.
Everyone has individual needs and characteristics. Do not assume confidence based on a person’s background.	Provide frequent opportunities for 1:1 discussion. Provide clear and tangible examples of the impact of the input of each group member throughout the project. Clarify everyone’s role from the outset, emphasising what unique insights everyone can contribute.
People have busy lives and fluctuating demands on their time - flexibility is important.	Provide a range of times and dates for each meeting. Use online polling software (or other) to streamline the availability process. Allow for additional 1:1 catch up sessions for group members who have missed a whole group meeting.
It is easy to get lost within busy intervention development projects - continued support to follow the progress of the project is essential.	Provide regular e-mails with key content about times, dates and links to activities. Facilitate ongoing communication between group sessions via a Whatsapp group (or similar). Provide presentation slides for each group meeting and always include a recap of the project to date at the start.
It is not just about the research project. It is also about enriching the wider professional and personal lives of all involved.	Conduct 1:1 discussions before the project commences, focusing on the motivations of each individual, what they are passionate about, and what they hope to get out of their experience. Throughout the project, allocate time to check in with each person about how the experience is going for them, and make changes if needed.

## Conclusions

In this paper our project steering group has reported and reflected on the impact of a partnership approach within the development of a complex, theory-based paediatric speech and language therapy intervention. Uniquely, this partnership approach involved bringing together a diverse range of professionals and people with lived experience within a unified project steering group. This approach to intervention design has resulted in the voice of each steering group member being woven into the intervention development process, evidenced by shared outputs such as papers and conference presentations. However, the professional and/or personal impact of being involved within the steering group has also been significant for each group member, with reflections highlighting that impacts go beyond supporting the intervention development itself. Each steering group member needed to feel safe when providing their honest and open reflections in this paper. We believe that this feeling of safety is the result of having worked together for a prolonged period and knowing that other group members are respectful and validating of each others’ opinions and experiences.

Although having a diverse range of people united within a single steering group has benefits, there are known challenges to this approach; group members may have disagreements, which can cause friction. This may be particularly relevant when considering the power dynamics within a mixed steering group of people with lived experience and professionals [37]. Although our opinions have varied due to our diverse backgrounds, we have been united by a shared goal, and there were no significant disagreements within our group. We believe this to be due to each group member being highly respectful of others’ opinions and having frequent opportunities to address differences in our sub-group brainstorming activities.

A further potential challenge of a unified approach is the additional resources needed to promote a safe, positive and collaborative working environment for each steering group member. For example, financial reimbursement is needed for the additional time involved for having individual meetings as well as whole group meetings. We recommend that researchers refer to the latest payment guidance for involving people in research [38] when planning for upcoming projects. We also would encourage researchers to consider the expertise of the researchers on their team; the academic lead for this project is an experienced speech and language therapist with a dual role as a researcher. Speech and language therapists bring unique expertise when supporting people with communication disabilities and the creation of accessible activities and written resources. The lead academic’s professional experience likely facilitated the inclusive environment in which our steering group activities took place.

We have described the short term benefits of involving individuals from a diverse range of backgrounds within a united intervention development steering group. However, we might consider how these may translate to longer term benefits for all involved. Taking part in research does not have to be a ‘one off’ experience, and positive experiences of research involvement can the foundations for future research involvement in other projects [39]. Steering group members now feel more confident about taking part in research, and we will take this confidence with us when we embark on other research projects in the future. There are also longer term impacts for the intervention we have developed. By having our sustained involvement throughout the development process, we have brought valuable insights which can help the intervention to be applicable to ‘real world’ contexts [39].

This is just one example in the growing field of case reporting in partnership-based approaches within healthcare research [40, 41]. Our principles and corresponding recommendations are complimentary to prior research findings, which indicate the importance of factors such as trust and communication within PPI [42, 43]. However, through our personal approach to reflection, we have been able to offer new insights into areas that have previously received little attention (e.g. ‘safety’). We hope that by applying our principles, future researchers can create positive, enriching contexts when uniting diverse individuals within the development of complex, theory-based interventions.

## Electronic supplementary material

Below is the link to the electronic supplementary material.

**Supplementary Material 1**: **Additional file 1**- Information sheet.

**Supplementary Material 2**: **Additional file 2**-Initial discussion checklist.

**Supplementary Material 3**: **Additional file 3**-Group principles.

**Supplementary Material 4**: **Additional file 4**-Role outline.

**Supplementary Material 5**: **Additional file 5**-PiiAF record card.

**Supplementary Material 6**: **Additional file 6**-Group reflections.

**Supplementary Material 7**: **Additional file 7**-Recommendations brainstorm.

## Data Availability

No datasets were generated or analysed during the current study.

## Abbreviations

SaLT: Speech and Language Therapist
DLD: Developmental Language disorder
SSD: Speech Sound Disorder
PPI: Patient and Public involvement
EDI: Equality, Diversity and Inclusion
PiiAF: Public involvement impact Assessment Framework

## Declarations

### Authors’ information

*Steering group members (public partners)*:

Dave Harford (DH) is a father of a child with lived experience of accessing NHS services for his son. He is an advocate for the speech and language therapy profession and works as a guest lecturer at Birmingham City University, where he shares his lived experiences with speech and language therapy students. He also engages in advocate work with the Royal College of Speech and Language Therapists (RCSLT).

Sophie Franks (SF) is an adult with DLD, with lived experience of accessing paediatric speech and language therapy services. She is a speech and language therapy advocate and in 2022 she was the recipient of the RADLD (Raising Awareness of DLD) outstanding achievement award. She also has professional experience of supporting children with additional needs within mainstream education settings.

*Steering group members (professional partners)*:

Emma Baker (EB) is a specialist speech and language therapist who works within a specialist provision for young children with DLD and/or SSD in Sheffield.

Laura Temple (LT) is a special educational needs teacher for the early years, providing outreach support for young children with speech and language needs in Brighton and Hove.

Meriem Amer-El-Khedoud (M A-K) is a university lecturer and specialist speech and language therapist who works in a specialist clinic supporting children with SSD and DLD in Tower Hamlets, East London. She also an advisor for the RCSLT regarding their equality, diversity and inclusion (EDI) initiatives.

Natalie Abdo (NA) is a specialist speech and language therapist who provides outreach support for young children with DLD and/or SSD in Brighton and Hove.

Patrycja Salimi-Tabar (P S-T) supports children with EAL (English as an Additional Language) with additional needs as part of the Ethnic Minority Achievement Service (EMAS) in Brighton and Hove. She also has lived experience of raising children within a tri-lingual household.

*The academic research team*:

Lucy Rodgers (LR) is a clinical academic speech and language therapist. The activities outlined in this paper are a part of her PhD, which aims to develop a new intervention for pre-school children with co-occurring features of DLD and SSD.

Nicola Botting (NB) is a researcher and university lecturer in the field of Developmental Psychology. She is a PhD supervisor for which the activities outlined in this paper are a part of.

Ros Herman (RH) is a researcher and university lecturer in the field of Speech and Language Therapy. She is PhD a supervisor for which the activities outlined in this paper are a part of.
