# Supplementary material for: “We’re all in it together”: uniting a diverse range of professionals and people with lived experience within the development of a complex, theory-based paediatric speech and language therapy intervention
Source: Res Involv Engagem. 2025 Jun 19;11:67. doi: 10.1186/s40900-025-00738-8 (PMC12180152; doi:10.1186/s40900-025-00738-8)
Supplement: Supplementary file 1 — Supplementary Material 1: Additional file 1- Information sheet. [file 40900_2025_738_MOESM1_ESM.docx]

**Additional file 1**

**Information sheet**

**Helping children with speech and language difficulties:**

**Our project steering group**


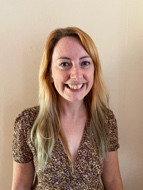


**About me**

Hi, I’m Lucy. I’m a **Speech and Language Therapist (SLT)** and PhD researcher at City University, London. The aim of the PhD project is to develop an **intervention** for pre-school children with features of both a **speech sound disorder** (SSD) and **developmental language disorder** (DLD) (*i.e. difficulties with their production of sounds as well as with learning new words).*

1. **Why is this project important?**

Pre-school children with this clinical presentation are at a **higher risk** of long-term literacy and communication needs when compared to children who have isolated speech *or* language difficulties. However, there are currently **limited intervention options** for targeting both speech AND language concurrently within NHS speech and language therapy services.


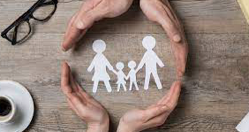


1. **What would this intervention ‘look like’?**

A SLT will be involved in delivering the intervention. However, we know that **children best develop their speech and language skills in situations that are meaningful to them**- including at home and when out and about with their family. Therefore, the intervention will involve the SLT working closely with the child’s family to improve communication in **everyday life**.

**The goal is to have a more detailed description of what the intervention looks like by the end of this project.**

**3) What is the purpose of the steering group?**

You will be a **diverse group** of **experts by experience**; including SLTs, parents, community representatives and adults who had speech and language therapy in childhood.


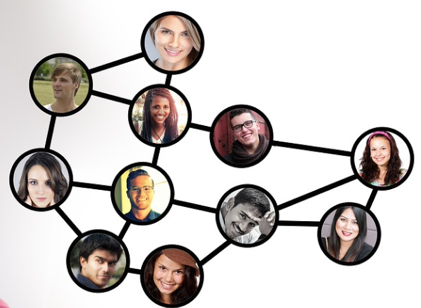


We need the intervention to be as **accessible** to as many people as possible. For example, we need to consider barriers faced by families from disadvantaged backgrounds and consider adaptions we might make for those who are ethnically and culturally diverse.

**I want to know, based on your experience, what matters to you and/or the community you work with.** Together with the current evidence base (existing research), this will then shape what the intervention looks like.

**4) What is the time commitment?**


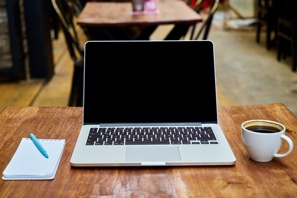


**We will meet online every 3-4 months, over the next 3 years**

- Mostly, meetings will last 1 hour
- I will send you **information to review** before each meeting (approx. 30 mins)
- We will have a **one to one chat** before each meeting (approx. 15-30 mins)

**This equates to approx. 2 hours every 3-4 months**

*(in the latter half of the project this will increase to* ***approx. 4 hours*** *every 3-4 months).*

**5) How will you help me to take part in the steering group?**

Meeting times/dates will be **flexible** and scheduled according to the preferences of the group.

If (on occasion) you are unable to attend a meeting, **alternatve arrangements** can be made (e.g. discussion over the phone).


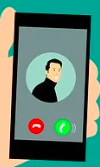

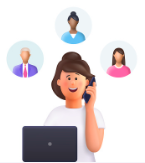

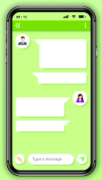


I will send out information beforehand re: how to join the group online. We can also **practice** this together if you want.

**6) What benefit will I get from being in the group?**

This is an opportunity to have **your say** in what a speech and language therapy intervention should look like for this group of children.

**I also want to ‘give back’ to you**. Before our first meeting, we will identify what you want to get out of the steering group experience. I will then endeavour to provide this when possible.

Your time and expertise are greatly valued and you will receive **payment**. The amount will depend on the stage we are at in the project, but as a guide you will receive **£25 in total for each 1-hour meeting and pre/post meeting activities**.

**Can I discuss this with you further?**

Email: -

Phone or text: -
