# Supplementary material for: “We’re all in it together”: uniting a diverse range of professionals and people with lived experience within the development of a complex, theory-based paediatric speech and language therapy intervention
Source: Res Involv Engagem. 2025 Jun 19;11:67. doi: 10.1186/s40900-025-00738-8 (PMC12180152; doi:10.1186/s40900-025-00738-8)
Supplement: Supplementary file 2 — Supplementary Material 2: Additional file 2-Initial discussion checklist. [file 40900_2025_738_MOESM2_ESM.docx]

**Additional file 2**

**Initial discussion checklist**

| **Area (based on UK standards for PI)** | **Y** | **Comments** |
| --- | --- | --- |
| Provide a brief overview of the intervention development phases |  |  |
| Explain why the steering group is needed |  |  |
| Explain why I’m approaching them, and what they could bring to the group |  |  |
| Ask ‘what motivates you, based on work and/or lived experience?’ |  |  |
| Discuss the role outline document |  |  |
| Ask- ‘what does meaningful involvement mean to you?’ |  |  |
| Clarify roles/responsibilities- what would they like to do? |  |  |
| Ask, “for me, a fulfilling steering group experience would look like…” |  |  |
| Ask, “by the end you will….”  (what would success look like to them) |  |  |
| Ask, “for me, a fulfilling steering group experience would look like…” |  |  |
| Discuss their prior knowledge of NHS SLT and how the I can help support knowledge development |  |  |
| Establish preferred methods of communication |  |  |
| What are their potential barriers- (internet, childcare, timings), and how I could help |  |  |
| Discuss experience and confidence with online platforms- zoom. Offer a practice with me. |  |  |
| Discuss their support network outside of the steering group. |  |  |
| Do they have any questions/comments? |  |  |
| What vouchers would they prefer |  |  |
| Discuss the shared principles document |  |  |
