# Supplementary material for: “We’re all in it together”: uniting a diverse range of professionals and people with lived experience within the development of a complex, theory-based paediatric speech and language therapy intervention
Source: Res Involv Engagem. 2025 Jun 19;11:67. doi: 10.1186/s40900-025-00738-8 (PMC12180152; doi:10.1186/s40900-025-00738-8)
Supplement: Supplementary file 3 — Supplementary Material 3: Additional file 3-Group principles. [file 40900_2025_738_MOESM3_ESM.docx]

**Additional file 3**

**Group principles**

Our Group Principles

Our group is a diverse range of people. This is great! However, it means we are different in various ways. We may not always agree with each other. We may present different solutions to the same problem. This *“diversity of opinion”* is important, and positive.

However, because of this, it’s important that we all agree with some basic principles:

1. **Everyone’s opinion matters**. Even if a suggestion cannot be taken forward, it should still be **valued** and acknowledged.

*Please note: The obvious exception to this is discriminatory comments around race, culture, sexuality, religion, gender, disability. This will not be tolerated under any circumstances.*

1. We need to allow space for **everyone** to express their views, on equal terms. **Every group member is an expert in their own right**.
2. Sometimes **listening** is as important as speaking. We need to listen to what others say, with an open mind. **We all have the potential to learn from each other**.

**I would like to hear from you. What do you think of these principles? Are there any you would add?**
