# Supplementary material for: “We’re all in it together”: uniting a diverse range of professionals and people with lived experience within the development of a complex, theory-based paediatric speech and language therapy intervention
Source: Res Involv Engagem. 2025 Jun 19;11:67. doi: 10.1186/s40900-025-00738-8 (PMC12180152; doi:10.1186/s40900-025-00738-8)
Supplement: Supplementary file 4 — Supplementary Material 4: Additional file 4-Role outline. [file 40900_2025_738_MOESM4_ESM.docx]

**Additional file 4:**

**Role outline**

The aim of this project is to develop a ‘draft’ of a pre-school speech and language intervention. It consists of 4 studies, over 3 years.

**About you**

1. You are a professional and/or expert by experience.
2. You are passionate about helping children with speech and language needs.
3. You have an awareness of the value of speech and language therapy.

**You will be involved with:**

1. Helping to decide **what goes into each study** *(e.g. questions for an online survey).*
2. Deciding what study findings are important to **‘real life’.**
3. **Bringing together** the findings from each study. We will then use this to inform our draft of the intervention.

**This involvement will look like:**

1. Attendance at a **steering group meeting**, every 3-4 months *(approx. one hour).* Please let me know as far in advance as possible if you cannot attend this meeting.
2. **Giving your availability** for these meetings via doodle poll.
3. **Looking at relevant documents** before the meeting *(approx. half an hour; this will increase to approx. 1 hour in the final 2 years of the project*).
4. Meeting with Lucy for a **1:1 chat** before each meeting (*approx. 15-30 minutes; this will increase to approx.1 hour in the final 2 years of the project).*
5. Keeping in **regular contact** via your preferred means.
6. Contributing to the development of **intervention materials** *(e.g. a session plan description)*, whilst understanding that **these are drafts and cannot be shared outside of our group yet.**

**What I will do to support you:**

1. I will arrange a **longer 1:1 meeting** with you if you are unable to attend the main steering group time/date.
2. I will communicate via your **preferred means.** This could be through email, text, voice call, video call, WhatsApp, social media messaging.
3. I will be **flexible about times/dates** for us to meet. We will schedule them according to when works best for you.
4. I will **reimburse your time.** This will be £25 per meeting and associated activities. This will increase when you spend more time on the project.
5. I will record your **personal goals** (i.e. what you want to gain from being a part of the group). I will then endeavour to support this throughout.
6. I will keep an **‘impact log’** to record the impact of our steering group work on this project. I will share this with you every 3-4 months.
7. Before each steering group meeting I will **share an agenda**, so you know what to expect
8. After each steering group meeting I will **share meeting notes**, which you can then add to/amend if needed
