# Supplementary material for: “We’re all in it together”: uniting a diverse range of professionals and people with lived experience within the development of a complex, theory-based paediatric speech and language therapy intervention
Source: Res Involv Engagem. 2025 Jun 19;11:67. doi: 10.1186/s40900-025-00738-8 (PMC12180152; doi:10.1186/s40900-025-00738-8)
Supplement: Supplementary file 5 — Supplementary Material 5: Additional file 5-PiiAF record card. [file 40900_2025_738_MOESM5_ESM.docx]

**Additional file 5**

**PiiAF record card**

| **Values**  *Based on:*  *Introductory 1:1s from 11/22-01/23 (impact log and notes)*  *First whole group meeting 9.1.23.* | **Despite the steering group being a mixed group of people with lived experience and various professionals, reasons for getting involved in the project overlap. These include:**  **1.Wanting to make a difference for future children/families**  **2.Wanting to expand learning + knowledge**  **3.Wanting to learn from others’ experiences within the steering group**  **Within the first whole group meeting, each group member talked about why speech and language therapy is important to them. This helped form a shared understanding- we are all ultimately here for the same reason: to improve the lives of children with communication needs.**  **Values for those involved in the phase 4 PPI- how to establish this within a relatively short timeframe? Incorporate into discussions with community links.**  **Impact assessment considerations:**  **Will need to look at to what degree Steering group members felt that their values were appreciated, and if their initial motivations for getting involved were met and nurtured.** |
| --- | --- |
| **Approaches to PPI**  *Introductory 1:1s 11/22-1/23 on how group members would like to be involved (impact log)*  *June 23 and Oct 23 whole group meeting notes + Miro boards-approach/action plan for phase 4a* | **There is a two pronged approach to PPI- firstly, a more formal steering group consisting of both professionals (with relevant EDI experience) and people with lived experience. Secondly, a wider group of families from diverse backgrounds (phase 4a). The PPI work at phase 4 would be less intensive for families (and hopefully therefore more accessible).**  **Due to the nature of the research question and emphasis on theory, intensive input is needed from a Steering group over 3 years. As the findings from each study phase come in, the Steering Group will work with the researcher to develop an intervention outline. They will also work with the researcher to refine the PPI strategy for phase 4a.**  **An EQIA assessment to identify barriers. These will be addressed through frequent reflection (checking in with Steering Group members in their 1:1s).**  **Impact assessment considerations:**  **Will need to look at positives and negatives of involving families at phase 4a, from their perspective- even if it is ‘successful’ in terms of intervention development was it a positive, empowering experience for them? Ethical duty. Was the approach for the Steering Group positive? Could it have been improved?** |
| **Research Focus and Study Design** | **The focus of this research is to identify the content, format and process of delivery for a theory-based speech and language therapy intervention (linguistic + behaviour change). The 4 phases are based on ‘key uncertainties’ in accordance with the MRC developing complex intervention guidance.**  **Families from low SES backgrounds, from the global majority and/or with limited English face additional barriers to accessing speech and language therapy interventions. Therefore, a second goal (woven into the intervention development process) is to have representation and involvement from families/those working with families from diverse backgrounds. Ultimately, we hope this will increase the accessibility of the intervention to all families.**  **Impact assessment considerations:**  **In addition to impacts for the intervention/project itself, we therefore also need to consider impacts for the people taking part in PPI. For example, how accessible were they? Any negative consequences? Would they do it again/take part in future PPI? Personal perspective. Phase 4a-Would need to consider the balance of power between them and who is asking them the questions- community link?** |
| **Practical Issues**  *Based on: Introductory 1:1s 11/22-1/23 (impact log)*  *1:1 check-ins, how’s it going for you? (impact log)*  *June 23 and Oct 23 whole group meeting notes + Miro boards-approach/action plan for phase 4a* | **Practical issues were identified in the initial 1:1 meetings, as well as solutions. These were revised in 1:1s. These included:**  **-arranging meeting times to suit everyone: doodle poll/giving lots of options, flexibility to meet up with researcher separately if unable to attend**  **-meeting reminders as people have busy schedules**  **-trying to keep meetings as concise as possible, whilst allowing space for gelling (meetings increased to 1.5 hours after feedback from June 2024 meeting)**  **Practical issues for phase 4a PPI explored in steering Group meeting/Miro brainstorms. Revised phase 4a protocol accordingly, to add in more flexibility for involvement.**  **Impact assessment considerations:**  **How successful were the practical solutions-were they feasible in practice? (opinion of Steering Group members). Phase 4a PPI- family views of the bespoke PPIE approach, what they thought was helpful, what may not have been helpful.** |
| **Identifying the Impacts of PPI in Research**  *Based on: Introductory 1:1s 11/22-1/23 (impact log)* | **Predominantly a theory-based intervention development project, so recording of impacts on the emerging theory of change for the intervention is important.**  **However, ethical duty to Steering Group members/phase 4 PPI families to make a positive experience for them which is not just ticking boxes. Go back to what Steering Group individuals said they personally wanted to get out of the process in our very first meeting- follow up on that. Phase 4 PPI, if it was a positive experience for them, it might encourage families to take part in similar projects again. Look at if their experience in this project has helped (or hindered) this, and why.**  **Unintended impacts can be recorded in the impact log as we go along and need to be incorporated into the evaluation- evaluation questions/strategy should be broad enough to capture them.** |
