# Supplementary figures and images for: “We’re all in it together”: uniting a diverse range of professionals and people with lived experience within the development of a complex, theory-based paediatric speech and language therapy intervention
Source: Res Involv Engagem. 2025 Jun 19;11:67. doi: 10.1186/s40900-025-00738-8 (PMC12180152; doi:10.1186/s40900-025-00738-8)

**Additional file 6**

**Group reflections**


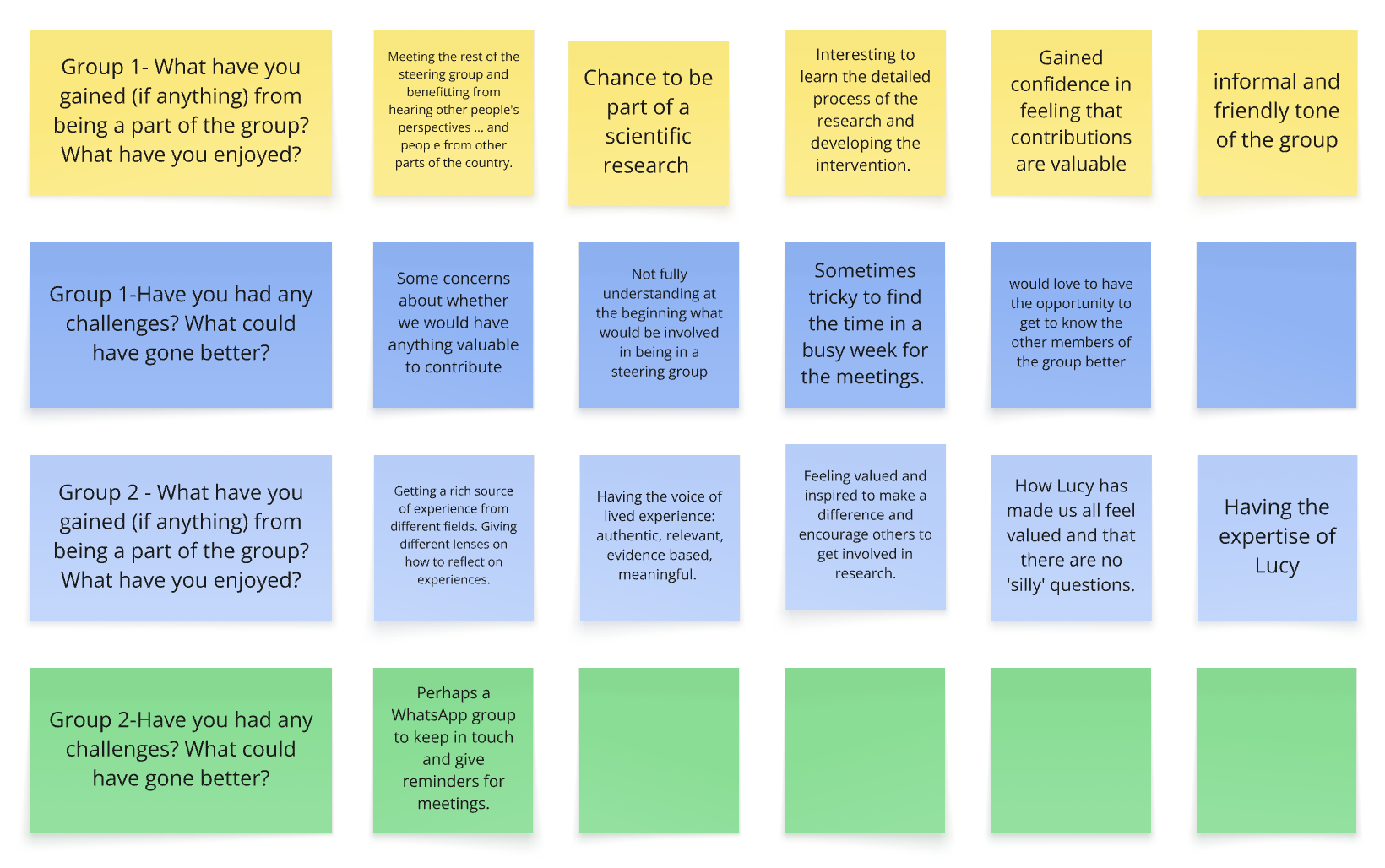

Supplement: Supplementary file 6 — Supplementary Material 6: Additional file 6-Group reflections. [file 40900_2025_738_MOESM6_ESM.docx]

**Additional file 7**

**Recommendations brainstorm**


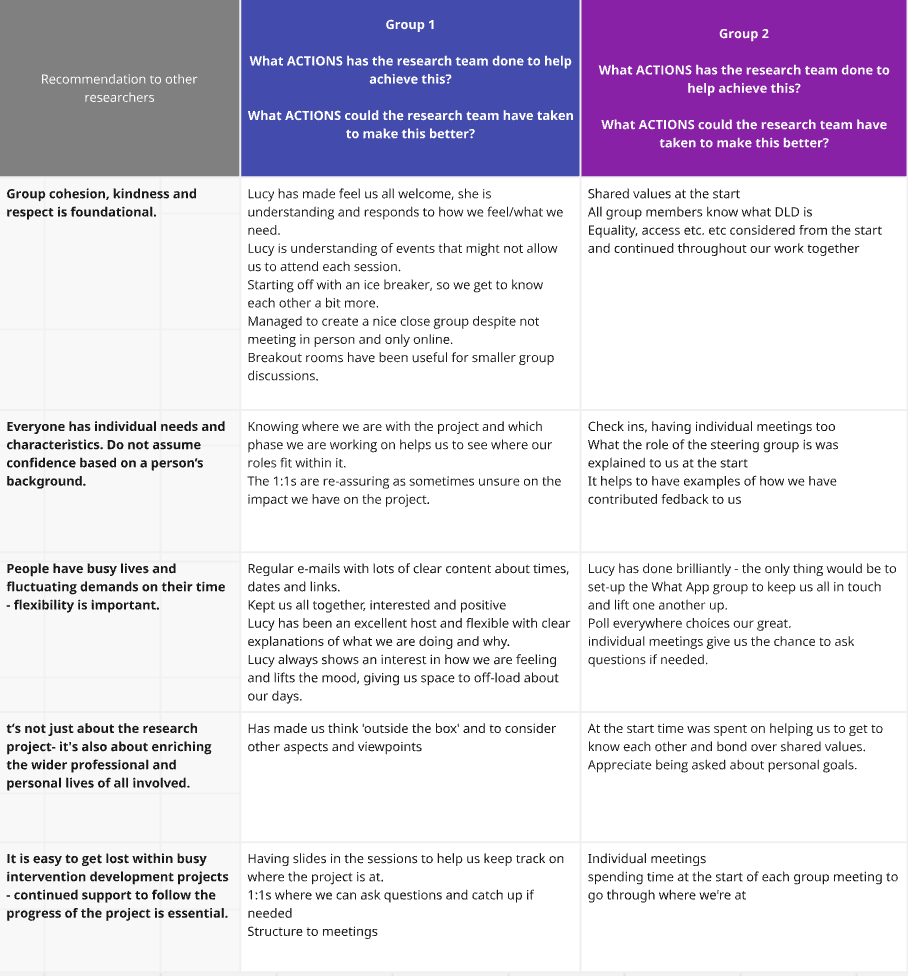

Supplement: Supplementary file 7 — Supplementary Material 7: Additional file 7-Recommendations brainstorm. [file 40900_2025_738_MOESM7_ESM.docx]
